# Comparative genomic and transcriptomic analysis of selected fatty acid biosynthesis genes and CNL disease resistance genes in oil palm

**DOI:** 10.1371/journal.pone.0194792

**Published:** 2018-04-19

**Authors:** Rozana Rosli, Nadzirah Amiruddin, Mohd Amin Ab Halim, Pek-Lan Chan, Kuang-Lim Chan, Norazah Azizi, Priscilla E. Morris, Eng-Ti Leslie Low, Meilina Ong-Abdullah, Ravigadevi Sambanthamurthi, Rajinder Singh, Denis J. Murphy

**Affiliations:** 1 Genomics and Computational Biology Research Group, University of South Wales, Pontypridd, United Kingdom; 2 Advanced Biotechnology and Breeding Centre, Malaysian Palm Oil Board, Kajang, Selangor, Malaysia; INRA, FRANCE

## Abstract

Comparative genomics and transcriptomic analyses were performed on two agronomically important groups of genes from oil palm versus other major crop species and the model organism, *Arabidopsis thaliana*. The first analysis was of two gene families with key roles in regulation of oil quality and in particular the accumulation of oleic acid, namely stearoyl ACP desaturases (SAD) and acyl-acyl carrier protein (ACP) thioesterases (FAT). In both cases, these were found to be large gene families with complex expression profiles across a wide range of tissue types and developmental stages. The detailed classification of the oil palm SAD and FAT genes has enabled the updating of the latest version of the oil palm gene model. The second analysis focused on disease resistance (R) genes in order to elucidate possible candidates for breeding of pathogen tolerance/resistance. Ortholog analysis showed that 141 out of the 210 putative oil palm R genes had homologs in banana and rice. These genes formed 37 clusters with 634 orthologous genes. Classification of the 141 oil palm R genes showed that the genes belong to the Kinase (7), CNL (95), MLO-like (8), RLK (3) and Others (28) categories. The CNL R genes formed eight clusters. Expression data for selected R genes also identified potential candidates for breeding of disease resistance traits. Furthermore, these findings can provide information about the species evolution as well as the identification of agronomically important genes in oil palm and other major crops.

## Introduction

The African oil palm, *Elaeis guineensis*, is a major global crop that can benefit considerably from the application of modern genomic advances. According to recent industry statistics, the annual global production of palm oil increased by almost 600% over the period 1990–2017, from 10.5 to 62.9 million tonnes [1,2]. In the global oils and fats sector, palm oil plays a crucial role in satisfying increasing demands as a key ingredient in edible oils for household food, ranging from manufactured ready meals to chocolate, and also as a feedstock for a wide range of non-food uses, such as for personal care and oleochemicals industries and, finally, as a renewable, carbon-neutral biodiesel fuel. While the availability of oil palm genomic sequences since the completion and publication of the oil palm genome sequence in 2013 [3] has facilitated the identification of genes involved in the regulation of important agronomic traits such as oil composition and disease tolerance, there are still considerable challenges in identifying which genes in large gene families should be targeted for manipulation via breeding.

In particular, the process of identification and characterization of full-length genes associated with agronomic traits can be complicated by the presence of closely related sequences that may have completely different functions. Another problem is that software-driven gene models and annotation methods can generate spurious entries in databases whereby sequences are incorrectly named and/or placed in the wrong gene family. For this reason, it is important that there is a large element of manual analysis and curation of genomic data, and that this is cross checked with transcriptomic and other wet-lab studies. In order to identify target genes regulating key traits in oil palm it is important that the sequenced genome is successfully mined in conjunction with transcriptome and field studies.

Oil composition is a key trait in the oil palm crop where its manipulation could enlarge its edible and non-edible uses and enable it to compete more effectively with high-oleate temperate oil crops such as soybean and rapeseed [4]. This is because an increasingly important aspect of fulfilling increasing consumer demand for edible oils is the optimisation of their nutritional status as so-called ‘healthy oils’. This predominantly relates to the amounts of lipophilic vitamins, such as vitamins A and E, the fatty acid composition of the particular vegetable oil, and whether it is a cold-pressed, ‘virgin’ oil or solvent and heat-treated, fully refined oil. In this respect non-refined red palm oil has distinctive advantages with high vitamin A and E contents, but the fatty acid profiles of both red and refined palm oils could benefit from optimization for some specific purposes, e.g. a more liquid oil for use in temperate countries.

Therefore the manipulation of fatty acid profiles through both conventional breeding and biotechnology strategies is a key focus of oil palm research [5]. In comparison to other main oil crops such as sunflower, rapeseed, soybean, maize and olive, oil palm fatty acid composition in most existing commercial varieties is characterised by an approximately 50:50 mixture of saturated and unsaturated fatty acids that is made up of 50% w/w of the saturated palmitic acid and stearic acids plus about 40% w/w oleic acid and 10% linoleic acids [4]. The relatively high saturate content of palm oil makes it very useful for the production and use of solid fats, such as spreads and chocolate products, in the food industry, but this can also limit its direct use as a fluid vegetable oil. In the future, the breeding of oil palm varieties with higher levels of oleic acid would enable the crop to expand into new market areas such as providing a direct feedstock for production of liquid vegetable oils for a range of edible and non-edible applications.

In terms of mesocarp oil quality, β-ketoacyl-acyl carrier protein synthase II (KASII), stearoyl-acyl carrier protein desaturase (SAD) and palmitoyl-acyl carrier protein thioesterase (FATB) are important components in the conversion of stearate to oleate in oil palm fruits [6,7]. Studies of SAD genes have also been reported in vegetables such as spinach and various brassicas, plus numerous important oil crops including safflower, soybean, jojoba and rapeseed [8]. In oil palm, two SAD genes have been cloned and their expression patterns in both kernel and mesocarp tissues reported [9,10]. Oilseeds such as rapeseed, soybean, sunflower, safflower and olive, can produce 75% or more oleic acid by non-transgenic methods and this value can increase to 89% for rapeseed and 90% for soybean [11]. Acyl-acyl carrier protein (ACP) thioesterase is another key enzyme required to produce a high oleic acid phenotype in oil palms. Two gene classes with slight differences in the derived protein sequences, FATA and FATB, have been well studied in both monocot and dicot species. These previous studies have reported on the activities and substrate specificities of the FAT enzymes from mesocarp tissues of oil palm [12] and the presence of three FATB genes and only one FATA in the oil palm genome [13,14].

In addition to research on improving oil quality, there are several other key agronomic traits that can have a major influence on oil production in the industry, one of the most important of these being disease resistance/tolerance. By far the most serious disease threat in the major oil palm-growing regions is basal stem rot caused by the fungal pathogen *Ganoderma boninense*. This is a major problem that leads to declining fruit yields in affected plants and the eventual death of trees across a wide area [15]. Resistance/tolerance to this and other important plant pathogens is mediated by numerous factors, but one of the most interesting groups is that of the so-called R (Resistance) genes [16]. One of the challenges in studying R genes is the complexity of their gene family and the presence of large numbers of closely related sequences, not all of which are necessarily involved in disease resistance *per se*. Classification of R genes is based on their domain organisation and a total of 9 distinct domains have been identified with as many as 16 gene classes [16]. A major R gene class is the coiled coil (CC) nucleotide-binding site (NBS)—leucine-rich repeat (LRR) Resistance genes (CNL), which are a subfamily of the NBS-LRR family proteins. Another class in this family is Toll/interleukin-1 receptor (TIR) NBS-LRR (TNL), but these genes have only been found in dicots to date [17]. In plant genomes as a whole, about 0.2–1.6% of all genes are from the NBS gene family and, while 0.5% of genes in the oil palm *pisifera* genome belong to this family, in the oil palm *dura* genomes the percentage of NBS family genes is probably higher [18–20].

In this study, we have focused on several genes encoding key members of the fatty acid biosynthetic pathway, notably SAD and FAT, and on putative R gene candidates involved in disease resistance in oil palm. For the latter genes, we aim to shed light on potential gene-for-gene interactions by the identification of specific R gene classes in order to assist our ongoing efforts to understand and control the molecular mechanisms of *Ganoderma* infection [21,22]. We have used a comparative genomics analysis for identification of orthologous genes from the oilseed species, *A*. *thaliana* and *Zea mays*, and have also compared predicted R genes in *E*. *guineensis* gene model sequences with two other monocot crop species, *Musa acuminata* and *Oryza sativa*. By using a comparative genomics approach, we hope to provide researchers with information to help in the identification and characterisation of the agronomically related genes of interest while also contributing to the understanding of oil palm evolution.

## Materials and methods

### Data collection for R genes and FA (fatty acid-related) genes

A total of 42 FA genes and 210 oil palm candidate R genes from the 26,059 representative gene models were used in this analysis [19]. Data from https://doi.org/10.1186/s13062-017-0191-4 was used in this paper and the sequencing data is from National Center for Biotechnology Information (NCBI) BioProject accession ID PRJNA345530. The protocol details are accessible at dx.doi.org/10.17504/protocols.io.mwcc7aw.

### Identification and global comparative genomics of oil palm gene model

Oil palm orthologous gene analysis against six plant gene models was performed using OrthoMCL2.0 [23] with default parameters. Table 1 shows the list of plants and the sources of data. In addition, to reconfirm that only the protein associated with R genes and FA genes was selected, a similarity search was done by using BLASTP [24] against the NCBI nr database with default parameters. Protein domain sequences were identified by various tools such as Pfam (http://pfam.sanger.ac.uk), InterPro (http://www.ebi.ac.uk/interpro/) and ScanProsite (http://prosite.expasy.org/scanprosite/) and NCBI CDD (http://www.ncbi.nlm.nih.gov/Structure/cdd/wrpsb.cgi).

**Table 1:** A list of gene model sources from six plant species.

Species	Sources
1. *O*. *sativa*	ftp://ftp.plantbiology.msu.edu/pub/data/Eukaryotic_Projects/o_sativa/annotation_dbs/pseudomolecules/version_7.0/all.dir/
2. *A*. *thaliana (TAIR 10)*	https://www.arabidopsis.org/download_files/Sequences/TAIR10_blastsets/TAIR10_pep_20110103_representative_gene_model_updated
3. *Phoenix dactylifera*	https://wcmq.app.box.com/s/yrt9lcgtlrkx56913r9yqbrn120waqb3
	http://qatar-weill.cornell.edu/research-labs-and-programs/date-palm-research-program/date-palm-draft-sequence
4. *M*. *acuminata*	http://banana-genome.cirad.fr/
5. *Z*. *mays*	http://phytozome.jgi.doe.gov/pz/portal.html#!info?alias=Org_Zmays

### Relationship between predicted oil palm genes and orthologs in monocots and *A*. *thaliana*

Protein members of the cluster sequences were aligned using two methods, ClustalW [25] and MAFFT version 7 [26]. In order to get an overview of the relationships between selected orthologous genes, phylogenetic trees were constructed using Molecular Evolutionary Genetics Analysis (MEGA7) [27] and tree from MAFFT [26].

### Gene ontology

766 single copy predicted genes were annotated using BLASTP hits to NCBI RefSeq plant protein database. Blast2GO [28] analysis was performed to assign the GO terms for these genes.

### Quantitative validation of single-copy genes via conserved orthologs using BUSCO

Benchmarking Universal Single-Copy Orthologs (BUSCO) (Gene set proteins assessment) [29] were used to validate the gene set of 766 single copy gene orthologs among species (*A*. *thaliana*, *Z*. *mays*, *P*. *dactylifera*, *M*. *acuminata* and *O*. *sativa*) identified from OrthoMCL and ClusterVenn [30].

### Expression profiles

Differential expression profiles of selected FA genes and CNL Class R genes in 22-tissue transcript libraries (BioProject PRJNA201497) were determined from the output of the Tuxedo suite pipeline (Bowtie2.1.0, TopHat2.0.9 [31], Cufflinks 2.2.1 [32], Cuffmerge 2.2.1, CuffDiff 2.2.1) mapped to the Pisifera 5 reference genome build assembly and linked to 26,059 gene model [19]. The R package library, Cummerbund, was used to plot heatmaps of significant differentially expressed R genes and FA genes generated by CuffDiff. RNA-seq data from kernel and mesocarp tissues were read mapped using the Tuxedo suite [33].

### Characterization of Acyl-ACP thioesterases

Experimental data from [14] was used in the characterization on the two classes of FAT genes in the acyl-ACP thioesterase subfamily. The sequences from subfamily A, B and C (plant acyl-ACP thioesterases) were downloaded from protein NCBI Genbank [34] database by using Batch Entrez.

## Results

### Distribution and function of orthologous groups

**Fig 1:**
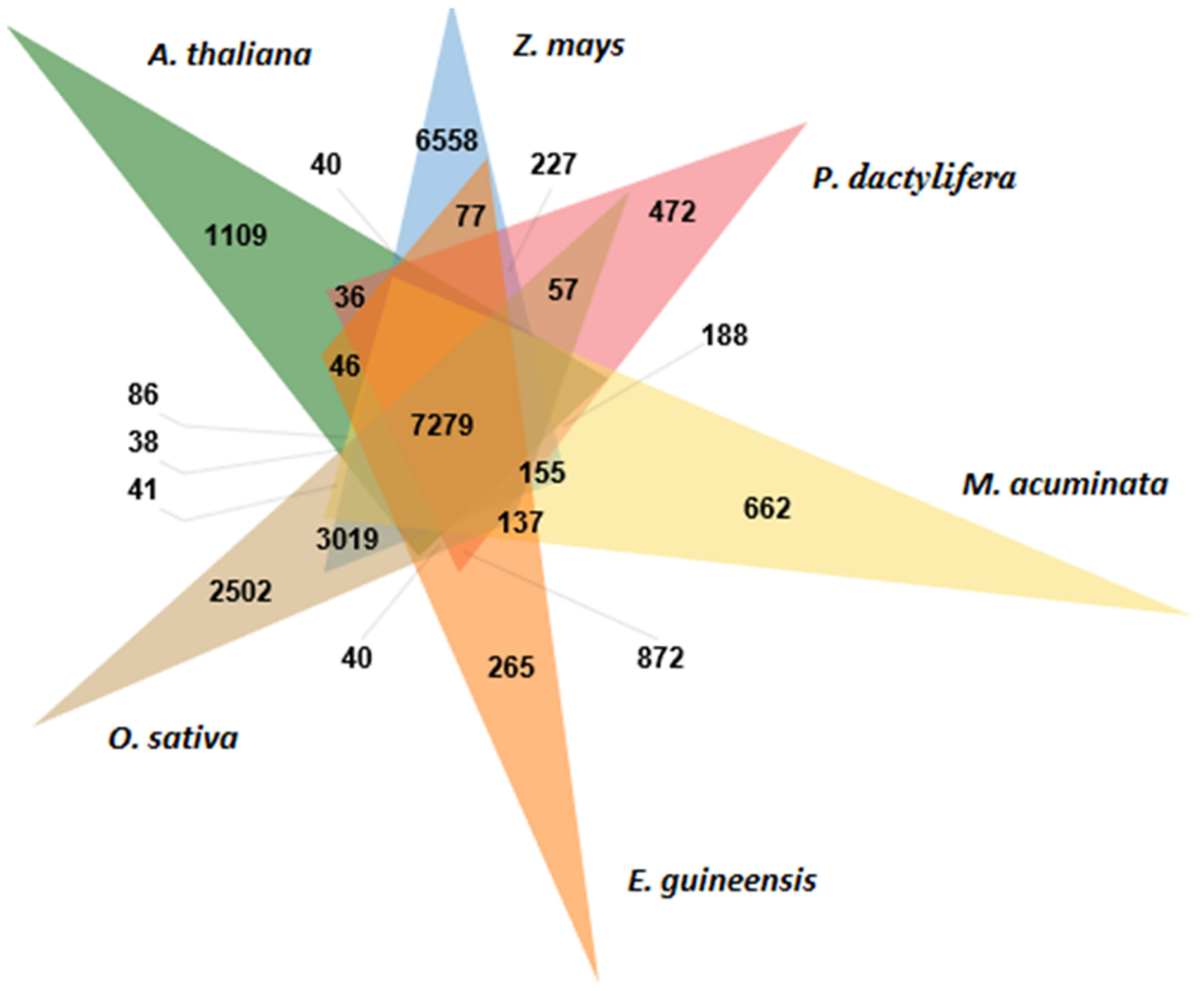
Venn diagram of orthologous groups in six plant genomes. Venn diagram generated by ClusterVenn from http://www.bioinfogenome.net/OrthoVenn/clustervenn.php.

**Fig 2:**
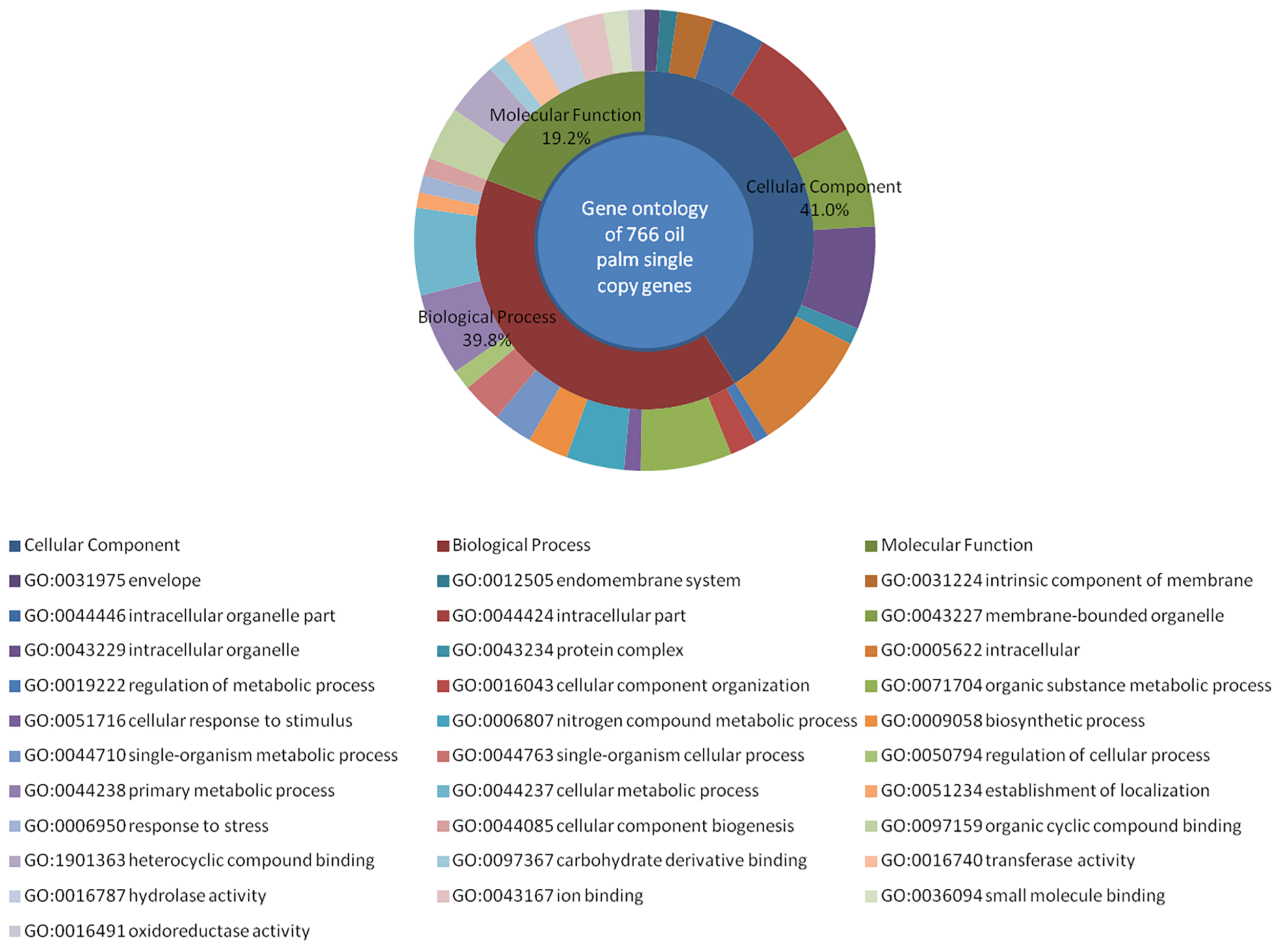
Gene ontology distribution (Level 3) of 766 oil palm putative single copy orthologous gene set. Details of full annotation for these sequences can be found in S1 Table.

Identification of orthologous genes among six genomes was performed using OrthoMCL and resulting in 7,279 clusters with a total of 94,337 ortholog sequences (*A*. *thaliana* = 12,681, *E*. *guineensis* = 13,273, *M*. *acuminata* = 15,639, *O*. *sativa* = 17,491, *P*. *dactylifera* = 12,020, *Z*. *mays* = 23,233). The results are summarised in Table 2. Fig 1 shows the distribution of shared and unique orthologous groups between six plant gene models. Oil palm single copy gene sequences were then used for BLASTP searches against RefSeq plant protein database with an e-value of 1e-5. Gene Ontology terms were mapped to this predicted set. Fig 2 shows the results for three functional categories: biological process, molecular function, and cellular component (S1 Table). The analysis showed that 2.9% of the total number of genes used in the model [19] were maintained as single copy genes after divergences. This result is important because such genes can be used as markers for genetic mapping and especially to anchor QTL associated with the traits of interest controlled by the genes. In order to validate the oil palm single copy orthologous gene set, BUSCO analysis was performed against the Embryophyta_odb9 plant lineage. Out of 766 sequences, only 20.3% were complete BUSCO sequences (291 complete and single-copy BUSCOs, one complete and duplicated BUSCOs, 15 Fragmented BUSCOs. Similar results were found with *A*. *thaliana* sequences where 310 complete BUSCO IDs were found. One of the reasons the results show relatively low numbers may be that BUSCO did not cover all date palm and oil palm species in the BUSCO group lineage. In addition, the present study is limited to six plant species, most of which are monocots that are quite closely related to oil palm.

**Table 2:** Number of genes and clusters identified between five different plant species and oil palm.

Species	Clusters (orthologous group)	Proteins in orthologous group (groups results)	Proteins in six taxonomy (7279 clusters)
*A*. *thaliana*	12,030	22,947	12,681
*E*. *guineensis*	12,444	22,189	13,273
*M*. *acuminata*	12,756	24,959	15,639
*O*. *sativa*	17,253	53,143	17,491
*P*. *dactylifera*	12,105	20,593	12,020
*Z*. *mays*	21,516	60,526	23,233

### Identification of relevant Stearoyl-ACP desaturase (SAD) genes and expression patterns in oil palm

**Fig 3:**
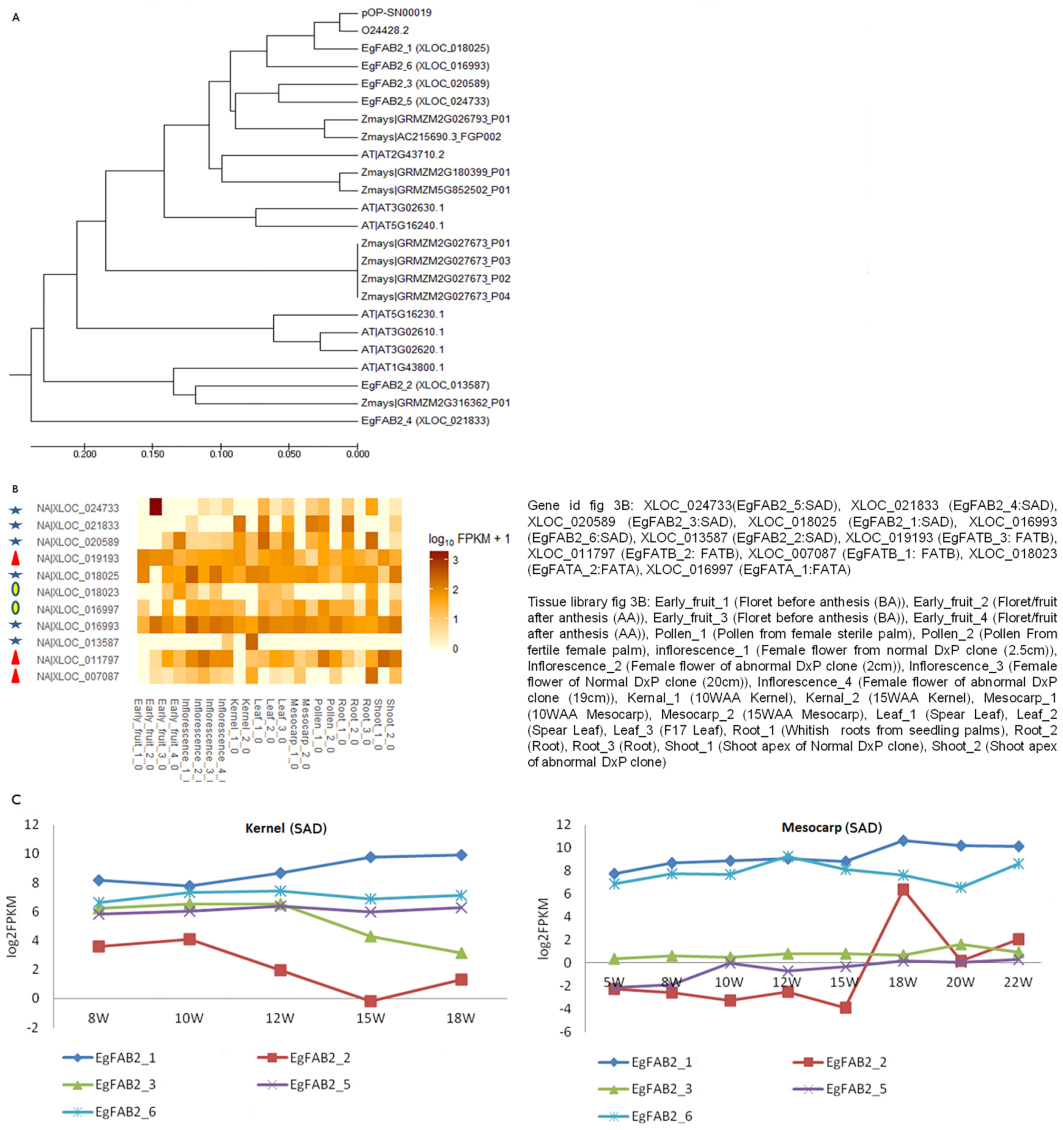
Phylogenetic and expression analysis of selected candidate SAD genes. (A) Evolutionary relationship of stearoyl-acyl carrier protein desaturase (SAD) in oil palm (*E*. *guineensis*), *A*. *thaliana* and *Z*. *mays*. Analyses were inferred using UPGMA method using MEGA6 software [38]. (B) Heatmap of SAD (Blue star) and FAT (Red triangle: FATB, yellow oval: FATA) gene expression in 22 transcriptome libraries. (C) Expression of SAD gene in kernel and mesocarp data.

When comparing predicted FA (fatty acid-related) genes in oil palm versus other plants such as *A*. *thaliana* and *Z*. *mays*, our analysis identified 29 orthologs in *A*. *thaliana* and 65 orthologs in *Z*. *mays*. The protein relationship of these three species was revealed as 17 clusters of 42 putative oil palm FA genes divided into 16 types of FA enzyme as shown in S2 Table which lists a number of putative orthologous FA genes in each organism. We identified two singletons, one related to FATB/SACPT (EgFATB_1) and one SAD (EgFAB2_3) gene. As the SAD genes play important roles in determining the ratio of saturated to unsaturated fatty acids in both plant membranes and storage lipids, we further investigated the other five SAD genes which are similar to *A*. *thaliana* and *Z*. *mays* (EgFAB2_1, EgFAB2_2, EgFAB2_4, EgFAB2_5 and EgFAB2_6). To investigate the evolutionary distance between them, a phylogenetic tree of the 24 orthologous genes was constructed (Fig 3A). Two oil palm sequences [9,10] which are supported by wet lab experimental data were BLASTed with six putative oil palm SAD genes. The pOP-SN00019 gene is 97.96% identical with O24428.2 and both sequences have similarities of more than 80% with EgFAB2_1, EgFAB2_3, EgFAB2_5 and EgFAB2_6. On the other hand, gene EgFAB2_2 clusters with Stearoyl-Acyl Carrier protein Δ9-Desaturase6 (SAD6) genes from both *A*. *thaliana* (AT1G43800.1) and maize (GRMZM2G316362). This SAD isoform is involved in the floral transition at the meristem 1 stage in *A*. *thaliana* [35] and it was recently [36] reported this SAD gene is also expressed at very high levels in maize kernels (endosperm plus embryo) tissues. The expression of SAD and FAT genes in the 22 transcriptome libraries is shown in the heatmap depicted in Fig 3B and more detailed expression in kernel and mesocarp during fruit development are shown quantitatively in Fig 3C. The six analysed SAD genes showed complex patterns of expression across all 22 libraries, consistent with the multiple roles of this key gene family in regulating the desaturation of C18 fatty acids in membrane and storage lipids and also in generating precursors for lipidic signalling molecules such as oxylipins [37]. Interestingly, the relative levels of SAD gene expression in the key lipid accumulating tissues of kernel and mesocarp were quite modest. The only SAD gene that showed a distinct upregulation in the developing mesocarp was XLOC_021833 (EgFAB2_4). The EgFAB2_2 (XLOC_013587) gene showed expression only in two tissues, namely kernel (15 week after anthesis [WAA] kernel), and inflorescence (female flower of abnormal DxP clone (19cm), while other SAD isoforms such as XLOC_018025 (EgFAB2_1) and XLOC_016993 (EgFAB2_6) showed higher levels of expression across a wider range of tissues and developmental stages. Based on Fig 3C, EgFAB2_2 shows distinct upregulation at 15 WAA in mesocarp which agrees with the fact that oil biosynthesis in the mesocarp starts at around 16 WAA. This same isoform also appears to increase in expression at 10 WAA and then decreases to its lowest level at 15 WAA in the kernel. This agrees well with the fact that oil synthesis in the kernel starts around 12 WAA and stops at 16 WAA, at which point oil synthesis starts in the mesocarp.

### Characterization and functional expression of oil palm thioesterase genes

**Fig 4:**
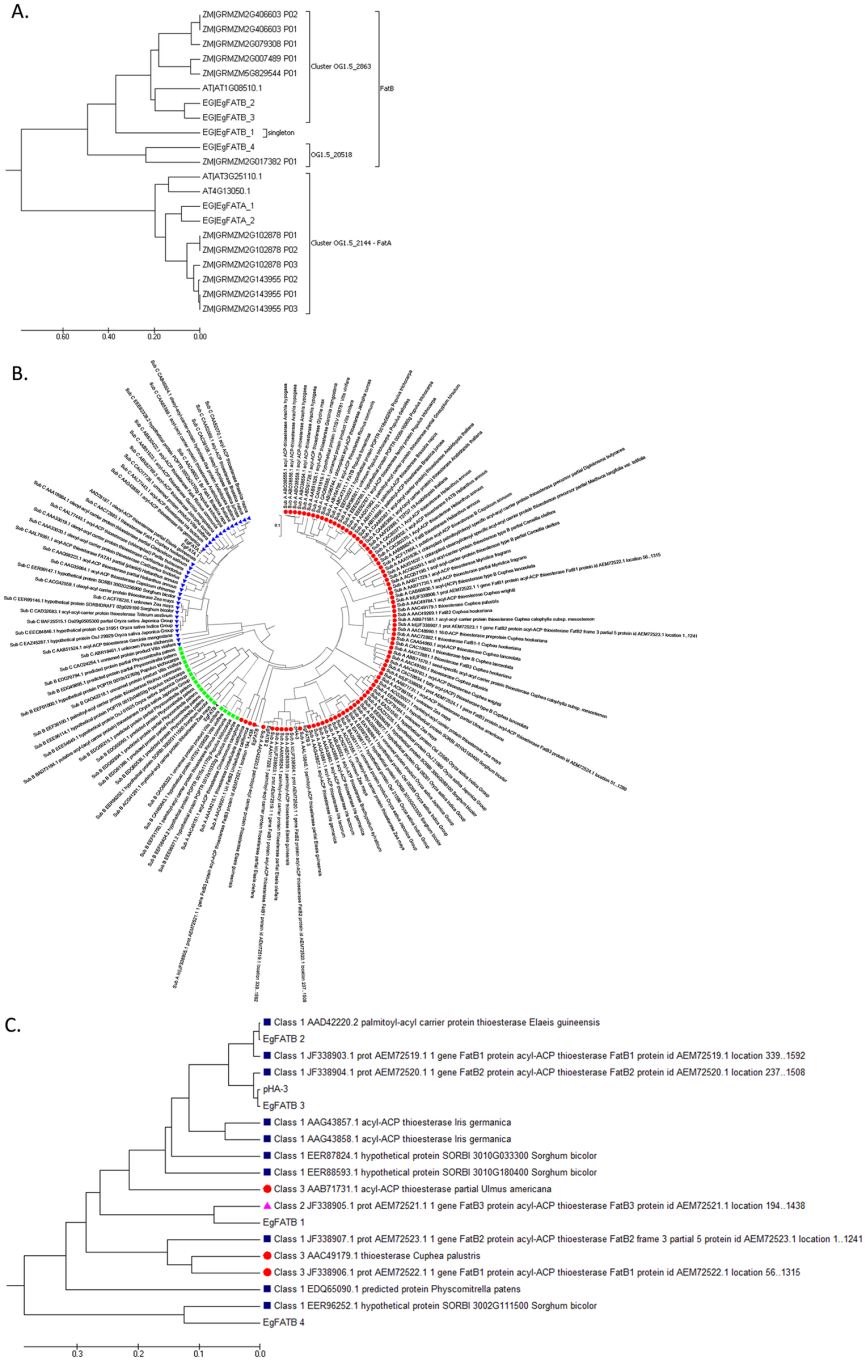
Phylogenetic and alignment analysis of selected candidate thioesterase (FAT) genes. (A) Phylogenetic tree of FATA and FATB sequences. (B) Phylogenetic tree of six putative oil palm acyl-ACP thioesterases that separated into subfamily A (red circle), subfamily B (green square) and subfamily C (blue triangle). A full list of sequences is available in S3 Table. (C) Classification of FATB genes. Classes 1, 2 and 3 are represented by a blue square, pink diamond and brown circle respectively. A full list of sequences is available in the S3 Table.

When comparing oil palm predicted FATA and FATB versus the *A*. *thaliana* and maize genes, our analysis identified one cluster for FATA (OG1.5_2144) plus two clusters (OG1.5_2863,OG1.5_20518) and one singleton for FATB. The tree in Fig 4A shows two main branches split between FATA and three clades represent three clusters of FATB. As reported in Fig 1 in [14], three subfamilies are categorised as plant acyl-ACP thioesterases. In subfamily A, 25% of the sequences have experimental evidence of expression/function and subfamily B currently has no supporting experiment data, while subfamily C is FATA group. Based on phylogenetic analysis, three FatB sequences (EgFATB_1, EgFATB_2 and EgFATB_3) is under Subfamily A. EgFATB_4 (FatB) is part of subfamily B group with EER96252.1 (*Sorghum bicolour*) which was previously characterized experimentally. Finally, subfamily C contained two FATA sequences (EgFATA_1 and EgFATA_2) (Fig 4B). FATB isoforms can be categorized into two groups where the first group is involved in the formation of C16:0 and is expressed in all plants and the second group is seed-specific and is involved in the formation of C8:0 –C14:0 medium chain fatty acids [39]. For the cluster analysis of four FATB clades according to their substrate specificity, various sequences from fatty acid composition data according to [14] were used. Three sequences (EgFATB_2, EgFATB_3 and EgFATB_4) are under Class I (with major activity towards C14 and C16 substrates), while EgFATB_1 was clustered under class II (broad range but with major activity towards C8 and C12 substrates) (Fig 4C). The alignments of the FATA and FATB oil palm and orthologs members are shown in S1 Fig respectively.

Fig 3B shows the expression of three FATB and two FATA genes in oil palm. Gene EgFATB_3 (NA|XLOC_019193) is expressed in all transcriptome libraries, while EgFATB_2 (NA|XLOC_011797) was expressed mainly in the mesocarp and in early stages of kernel development at 15WAA. Gene EgFATB_1 (NA|XLOC_007087) was highly expressed in 15WAA kernels and in 10WAA mesocarp tissues, while EgFATB_4 was not expressed in either tissue. One FATA, EgFATA_2 (NA|XLOC_018023) was not expressed in fruit tissues, while EgFATA_1 (NA|XLOC_016997) was expressed in 10WAA and 15WAA mesocarp and in 10WAA kernels. The FATA expressed in the mesocarp is 98% similar to EgFATA_1 and is responsible for cleaving oleoyl-ACP to release oleic acid in palm oil [13]. Other factors in addition to FATA that are responsible to 39% oleic acid levels typically found in palm mesocarp oil include KASII, FATB and SAD.

**Fig 5:**
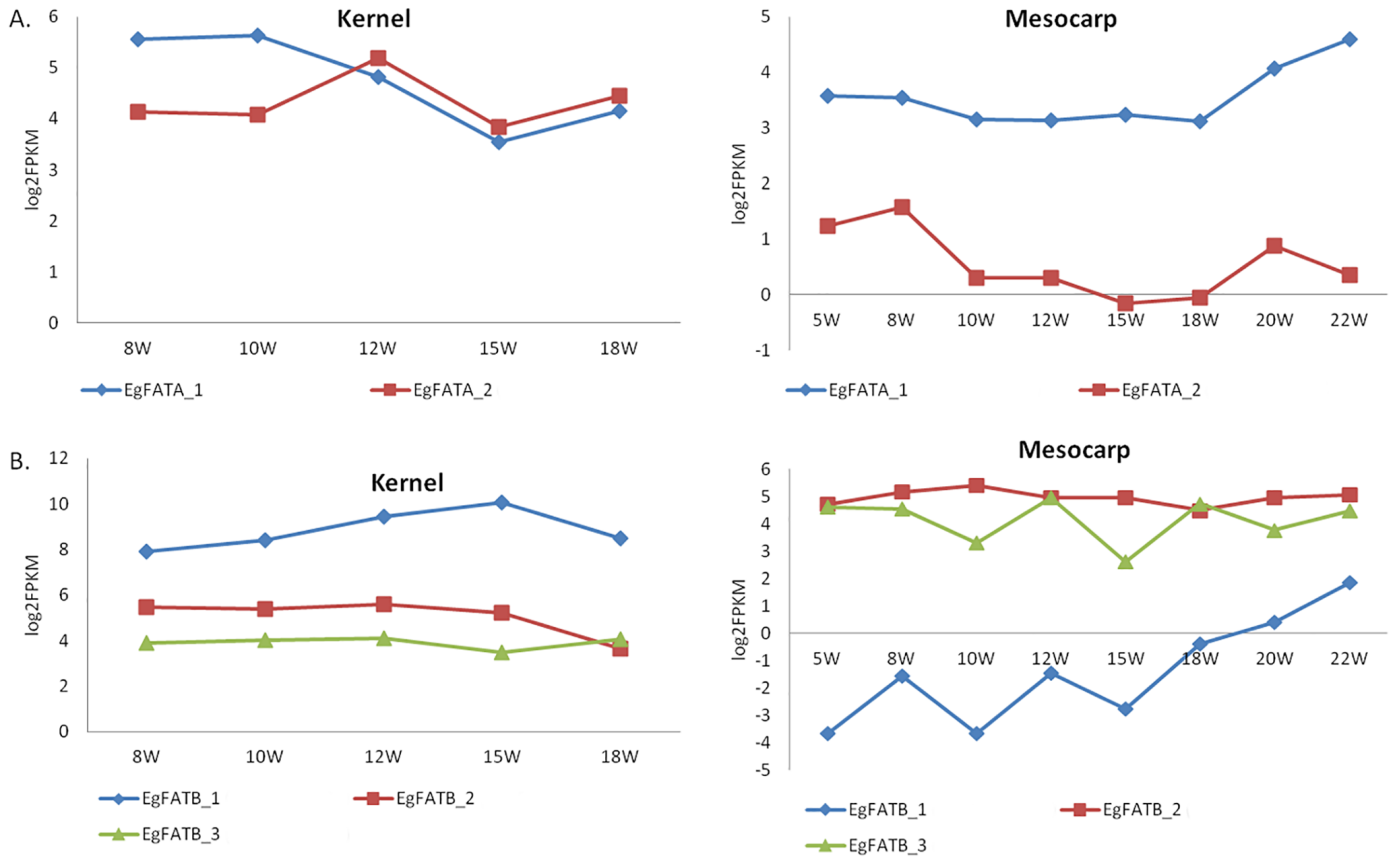
Thioesterase expression profile. (A) FATA expression profile in kernel and mesocarp for different time points in weeks after anthesis (WAA). (B) FATB expression profile in kernel and mesocarp for different points in weeks after anthesis (WAA).

The FAT gene expression data in Fig 3B and in Fig 5 are interesting because in storage tissues the FATA and FATB enzymes play important roles in regulating the cleavage of fatty acyl-ACP esters and in the channelling of the fatty acids towards triacylglycerols rather than further metabolism via elongation and desaturation. One of the key targets of oil palm manipulation is to downregulate the particular FAT gene that actively cleaves palmitoyl-ACP and thereby contributes to the relatively high levels of palmitic acid in palm oil [12–14,40]. As expected, in kernel tissues the expression of all five analysed FAT genes remained relatively constant throughout development. The EgFATB_4 gene was partially mapped to the transcriptome data and detected at a very low level of expression in the mesocarp and kernel time course data. Surprisingly there was no clear candidate FAT gene that was specifically upregulated during mesocarp development (Fig 5). As shown in Fig 5 (lower right panel), the transcript abundance of gene EgFATB_1 increased steadily during mesocarp development but it was still lower than the expression levels of the other two FATB genes, which remained relatively constant over the same time period. This may indicate that the control over fatty acid flux towards palmitate accumulation in storage triacylglycerol (TAG) does not primarily reside in the expression levels of the relevant FAT gene(s) *per se*.

### Identification and expression pattern of CNL class resistance (R) genes

When comparing the 210 predicted R genes in the oil palm genomes versus those in the published banana and rice genomes, our analysis identified 634 orthologous genes in 37 clusters (*M*. *acuminata* = 164, *O*. *sativa* = 329). Of the 210 oil palm putative R genes, 141 orthologs (versus banana and rice) were classified into four different classes (Kinase = 7, CNL = 95, MLO-like = 8, RLK = 3 and Others = 28). Using the canonical NBS, and LRR motifs, the Resistance gene family of oil palm can be divided into two subfamilies; TIR and non-TIR. Based on the N-terminus structure, TIR-NBS-LRR contained both toll and interleukin-1 receptor domains, while most of the non-TIR-NBS-LRR had a coiled-coil domain. In the oil palm genome, eight clusters are categorised as putative CNL R genes. Most of the candidate R genes were previously classified based on BLAST results as TIR-NBS-LRR are now reclassified as non-TIR-NBS-LRR a genes. Only two sequences (Eg_rgh_cnl_51, Eg_rgh_cnl_94) could not be confirmed as non-TIR-NBS-LRR genes due to the absence of the coiled-coil and tryptophan (W) motifs at the kinase 2 domain. Protein relationships showed that Eg_rgh_cnl_51 is similar to GSMUA_Achr3P27810_001 as part of the second largest cluster with 87 orthologous sequences. Meanwhile, Eg_rgh_cnl_94 is from a large cluster with 32 oil palm sequences, plus 49 orthologous sequences from *O*. *sativa* and 34 from *M*. *acuminata*.

**Fig 6:**
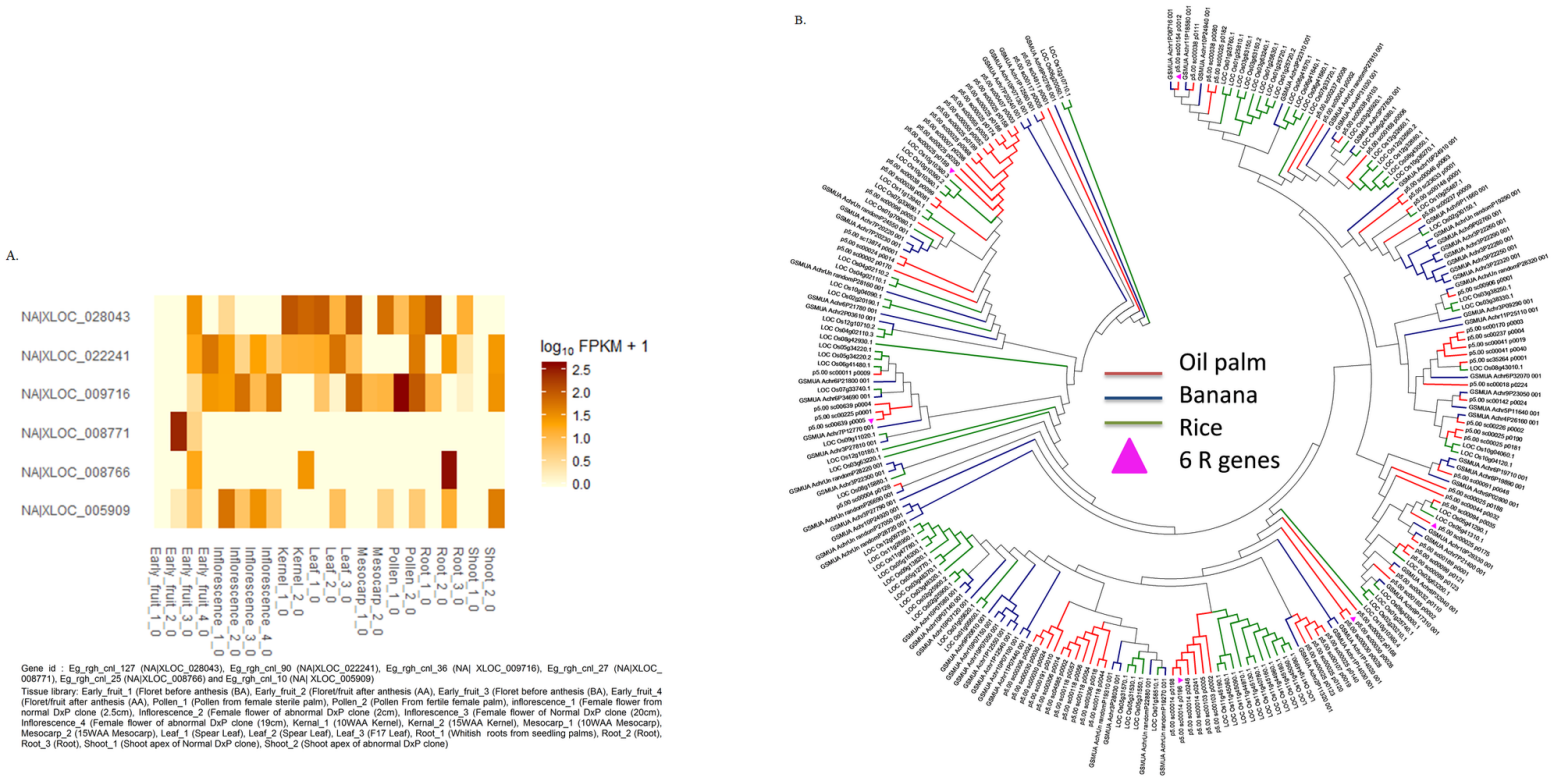
Phylogenetic and expression analysis of selected candidate R genes. (A) Heatmap of the six R genes expressed in 22 oil palm transcriptome libraries. (B) Phylogenetic tree between *M. acuminata* and *O. sativaorthologs* with eight oil palm R genes constructed using Mega7.

Transcriptome data showed that expression of the selected putative R genes varies greatly from one tissue to another (Fig 6A). The Eg_rgh_cnl_27 (NA|XLOC_008771) resistance genes showed highly upregulated expression patterns in the early stage of fruit developments (Floret/fruit after anthesis). Other interesting results showed that Eg_rgh_cnl_25 (NA|XLOC_008766) was expressed in kernel [15WAA] and root and the isoform (TCONS_00012090: 472bp) hit to RRP8-like disease resistance. Four clusters with 232 orthologs members which consist of six expressed R genes were used to built phylogenetic trees. From the tree shown in Fig 6B, the branch distributions clearly support the close relationship between oil palm and banana and a more distant relationship with the other analysed species.

## Discussion

Clustering of orthologs across the six selected crop and model plant species offers a potentially rapid and efficient approach for characterization of gene families in the oil palm genome. Comparative genomics can greatly facilitate gene curation and gene prediction including identification of transcription factor and promoter motifs [41,42]. Compared to the other five plant species, there was a particularly high number of conserved protein sequences that was shared between banana and oil palm (10,337), which is consistent with the close evolutionary relationship between their genomes [3]. Another interesting result from gene ontology (GO) annotations was that out of the 2.9% of total oil palm genes that were present as single copies, no fewer than 128 sequences were characterised under ‘cellular aromatic compound metabolic process’ terms (biological function class) of which 39 genes were listed as being involved in ‘aromatic compound biosynthetic process’. This list of genes provides useful candidates to enable us to better understand the biosynthetic pathways of aromatic compounds, and especially shikimic acid, which is an important metabolite in the formation of oil palm phenolics, which are an additional class of useful compounds that are being studies for their potential nutritional and medical applications [43,44].

The optimisation of fatty acid content in oil palm is a key goal for increasing edible oil quality for the benefit of the overall oil palm market [11]. The SAD and thioesterase gene families in oil palm are believed to play important roles in controlling the desaturation of stearic acid and the accumulation of specific C16 or C18 chain lengths in mesocarp storage lipids [45] but in both cases these are large gene families and it is important to ascertain which isoforms are specifically involved in storage lipid accumulation. Interestingly, EgFAB2_4, which was previously defined as a singleton [19] has now been found to be part of a group with other five other SAD genes. The results obtained after *M*. *acuminata*, *Z*. *mays* and *P*. *dactylifera* were added in the orthoMCL analysis. It has been reported that nine surveyed plant SAD genes shared an average 80% sequence identity [46]. In line with this finding, multiple sequence alignment analysis showed that the oil palm SAD genes also had high similarity scores (> 70% amino acid sequence identity) with those of *Z*. *mays* and *A*. *thaliana* (S1 Fig). Although EgFAB2_3 remain as a singleton, this sequence is similar to EgFAB2_5 (Fig 3A). Fig 3A shows that EgFAB2_1 is in same clade as sequences from NCBI accession numbers O24428.2 and pOP-SN00019 [9]. Surprisingly, EgFAB2_6 with 89% similarity to EgFAB2_1 was also identified in this clade.

Characterization and identification of the two thioesterases classes, FATA and FATB, is important in understanding the control mechanisms that determine the chain length and level of saturation in palm mesocarp oil. More broadly, however, it can be difficult to distinguish between putative FATA and FATB isoforms because of the high sequence similarity between these two enzymes [47]. In *Cuphea* seeds, which accumulate short-to-medium chain fatty acids, two acyl-acyl carrier protein thioesterases were identified, one of which was more specific to shorter chain acyl groups while the other was more active with long-chain fatty acids [48]. Subsequent evolutionary studies of other plant species revealed there are several differences between the sequences of these two thioesterase classes [49]. In several oilseed species, including *A*. *thaliana* and *B*. *napus* the genetic modification of FATB genes has resulted in increased levels of palmitic acid in the storage TAG [50]. While previously in the oil palm genome, four acyl-ACP thioesterases were identified [13], we have identified six oil palm acyl-ACP thioesterases, that include one additional FATA and FATB [19]. For FATB, the gene annotated here as EgFATB_2 is similar to EgFATB1 from [13], and EgFATB_3 to EgFATB2 [13] and EgFATB_1 to EgFATB3 [13]. For FatA, EgFATA_1 is similar to EgFATA [13]. Therefore the new FATB gene found in this study is EgFATB_4 and the new FatA is EgFATA_2. According to the phylogenetic tree shown in Fig 4B, the new EgFATB_4 gene clusters with orthologs from *Sorghum bicolor* (EER96252.1) and *Z*. *mays* (ACG41291.1) under subfamily B (green squares). The two FATA genes are located in subfamily C (blue triangles) together with clade AAD28187.1. These results suggest that the new FATB gene belongs to a group for which there is no wet lab conformational evidence at present. The characterization of SAD, FATA and FATB gene families will help to elucidate the gene regulatory networks involved in fatty acid composition and oil content in oil palm. For example, in a recent study in pecan, expression of these genes was correlated with levels of oleic acid in storage oil [51].

Identification of R genes can help to improve screening for disease resistance/tolerance for major for oil palm pathogens such as *Ganoderma boninense*, which cause major yield losses in the crop [15]. These analyses also improve our knowledge of the number of genes present in such large gene families in the oil palm genome in a way that informs evolutionary studies. Identification of R genes is important as they are expressed in early stages of the oil palm defence mechanism from three major diseases (Fusarium wilt, bud rot and basal stem rot) and may be used to identify and treat infected plants. Similar diseases have been found in banana and many disease resistance genes, both putative and demonstrated, have been also detected in rice, which provides a good model for comparative studies of the largest class of R genes [52,53]. Some oil palm resistance genes were initially identified from GeneThresher methylation data [54], but this number has increased to 210 genes based on our latest oil palm gene models [19]. R genes have been characterised into five different classes and recently the number increased to 16 in the 39 most important agricultural plant species [55,56]. This classification of R genes is based on the domain organization and, in the case of CNL R genes, the presence of leucine rich repeat (LRR) and coiled-coil (CC) motifs is an absolute requirement.

We found a total of 61 R gene candidates with a predicted coiled-coil-NBS-LRR (CC-NBS-LRR) domain, while 32 are characterized by the presence of the last residue W (Tryptophan) at the kinase 2 site within the NBS domain [17]. In plant genomes as a whole, about 0.2–1.6% of all sequences are from the NBS gene family while 0.5% of genes in the oil palm *pisifera* genome belong to this family and in the oil palm *dura* genomes the percentage of NBS family genes is probably even higher [18–20]. In this study, we found 95 CNL R genes with 77 orthologous genes in banana and 137 in rice. The number of orthologs in oil palm and banana is very similar. While oil palm, banana and rice are all monocots, they belong to different orders and the Zingiberales (banana) are much more closely related to Arecales (oil palm) than to Poales (rice) [57]. The phylogenetic tree based on derived protein sequences from our identified R genes (Fig 6) shows the close relationship between these three taxa. Four out of six R genes expressed in 22 oil palm transcriptome libraries were mapped to the oil palm multi-parental population genetic map with one gene each in chromosomes one, two and twelve [58]. Two further putative R genes, which could not be confirmed as non-TIR-NBS-LRR genes, were found on chromosomes two and six [58].

In conclusion, we have characterised the members of three large gene families in oil palm with respect to their sequences, chromosome locations, phylogeny and expression profiles in a wide range of tissues and developmental stages. In all cases the gene family members show complex patterns of expression and regulation that underlie their multiple roles in the plant. These comparative genomics results reveal important information about the species evolution as well as the identification of sequences that are unique to a particular species. Additionally, these findings contribute valuable resources in the form of candidate marker genes that can be tested by breeders as part of screening for agronomic traits at early stages of trials. In particular this work will assist our ongoing efforts to identify specific targets for breeders seeking to improve key traits such as fatty acid quality and disease resistance/tolerance in oil palm.

## Supporting information

S1 Table Gene list and gene annotation. (XLSX)

S2 Table List of orthologous genes identified in Arabidopsis and maize genes. (PDF)

S3 Table Three subfamilies and classification of FATB. (PDF)

S1 Fig Multiple sequence alignments of FATA, FATB and SAD. [A] Alignment of two oil palm FATAs with orthologs from *A*. *thaliana* and *Z*. *mays*. [B] Alignment of two oil palm FATBs with orthologs from *A*. *thaliana* and *Z*. *mays*. [C] Protein sequence alignment of the orthologous cluster OG1.5_1281 of stearoyl-acyl carrier protein desaturases (SAD) aligned using MUSCLE to produce more than 70% identity between oil palm and *A*. *thaliana* and maize proteins.
(PDF)
