# Supplementary material for: Comparative genomic and transcriptomic analysis of selected fatty acid biosynthesis genes and CNL disease resistance genes in oil palm
Source: PLoS One. 2018 Apr 19;13(4):e0194792. doi: 10.1371/journal.pone.0194792 (PMC5908059; doi:10.1371/journal.pone.0194792)
Supplement: S2 Table — (PDF) [file pone.0194792.s002.pdf]

LIST OF ORTHOLOGOUS GENES IDENTIFIED IN ARABIDOPSIS AND MAIZE GENES

**Enzyme**	**Group**	***A.thalina***	***Z.mays***	***E.guineensis***
1. Multifunctional form of Acetyl-CoA carboxylase	OG1.5_2696	2	5	p5.00_sc00054_p0048
	OG1.5_5713	1	3	p5.00_sc00095_p0046
2. Multisubunit form of Acetyl-CoA carboxylase	OG1.5_7074	1	0	p5.00_sc00020_p0225, p5.00_sc00017_p0152, p5.00_sc00197_p0009
3. Multisubunit form of Acetyl-CoA carboxylase	OG1.5_7056	2	0	p5.00_sc00011_p0007, p5.00_sc00227_p0009
4. Multisubunit form of Acetyl-CoA carboxylase	OG1.5_9489	1	0	p5.00_sc00019_p0245, p5.00_sc00077_p0033
5. Malonyl-CoA:ACPtransacylase	OG1.5_7628	1	2	p5.00_sc00007_p0027
6. β-ketoacyl-ACP synthase III	OG1.5_7075	1	1	p5.00_sc00035_p0050, p5.00_sc00049_p0018
7. β-ketoacyl-ACP synthase I<br>8. β-ketoacyl-ACP synthase II	OG1.5_1405	2	10	p5.00_sc00001_p0491,p5.00_sc00004_p0248,p5.00_sc00064_p0074,p5.00_sc00064_p0077,p5.00_sc00004_p0242,p5.00_sc00036_p0047,p5.00_sc00035_p0142
9.β-ketoacyl-ACP reductase	OG1.5_2864	1	5	p5.00_sc00006_p0025, p5.00_sc00034_p0180
10. β-hydroxyacyl-ACP dehydrogenase	OG1.5_4080	2	2	p5.00_sc00012_p0164, p5.00_sc00271_p0017
11. Enoyl-ACP reductase	OG1.5_4279	1	3	p5.00_sc00037_p0126, p5.00_sc00131_p0012
12. Stearoyl-ACP desaturase	OG1.5_1397	7	9	p5.00_sc00147_p0015, p5.00_sc00229_p0026, p5.00_sc00091_p0032, p5.00_sc00051_p0096, p5.00_sc00078_p0031
	singletons			p5.00_sc00125_p0015
13. Oleoyl-Phosphatidylcholine desaturase	OG1.5_1952	1	8	p5.00_sc00037_p0124, p5.00_sc00131_p0010

14. Linoleoyl-Phosphatidylcholine desaturase	OG1.5_1916	3	5	p5.00_sc00003_p0184, p5.00_sc00033_p0032, p5.00_sc00121_p0039
15. Acyl-ACP thioesterase	singletons			p5.00_sc00018_p0248
	OG1.5_2863	1	5	p5.00_sc00039_p0052, p5.00_sc00104_p0027
	OG1.5_20518	0	1	p5.00_sc00052_p0027
16. Oleoyl-ACP thioesterase	OG1.5_2144	2	6	p5.00_sc00078_p0041, p5.00_sc00091_p0027
