# Supplementary material for: Comparative genomic and transcriptomic analysis of selected fatty acid biosynthesis genes and CNL disease resistance genes in oil palm
Source: PLoS One. 2018 Apr 19;13(4):e0194792. doi: 10.1371/journal.pone.0194792 (PMC5908059; doi:10.1371/journal.pone.0194792)
Supplement: S3 Table — (PDF) [file pone.0194792.s003.pdf]

**Subfamily A**

| Acc. No | Description |
|---|---|
| AAA34215.1 | AAA34215.1 thioesterase [Umbellularia californica] |
| AAB51525.1 | AAB51525.1 acyl-ACP thioesterase [Garcinia mangostana] |
| AAB71729.1 | AAB71729.1 acyl-ACP thioesterase [Myristica fragrans] |
| AAB71730.1 | AAB71730.1 acyl-ACP thioesterase, partial [Myristica fragrans] |
| AAB71731.1 | AAB71731.1 acyl-ACP thioesterase, partial [Ulmus americana] |
| AAB88824.1 | AAB88824.1 FatB thioesterase [Helianthus annuus] |
| AAC48990.1 | AAC48990.1 16:0-ACP thioesterase preprotein [Cuphea hookeriana] |
| AAC49001.1 | AAC49001.1 Uc FatB2 [Umbellularia californica] |
| AAC49151.1 | AAC49151.1 acyl-ACP thioesterase [Cinnamomum camphora] |
| AAC49179.1 | AAC49179.1 thioesterase [Cuphea palustris] |
| AAC49180.1 | AAC49180.1 thioesterase [Cuphea palustris] |
| AAC49269.1 | AAC49269.1 FatB2 [Cuphea hookeriana] |
| AAC49783.1 | AAC49783.1 acyl-ACP thioesterase [Cuphea wrightii] |
| AAC49784.1 | AAC49784.1 acyl-ACP thioesterase [Cuphea wrightii] |
| AAC72881.1 | AAC72881.1 thioesterase FatB3 [Cuphea hookeriana] |
| AAC72882.1 | AAC72882.1 thioesterase FatB1-1 [Cuphea hookeriana] |
| AAD01982.1 | AAD01982.1 palmitoyl-acyl carrier protein thioesterase, partial [Gossypium hirsutum] |
| AAD42220.2 | AAD42220.2 palmitoyl-acyl carrier protein thioesterase [Elaeis guineensis] |
| AAF22899.1 | AAF22899.1 T27G7.19 [Arabidopsis thaliana] |
| AAG43857.1 | AAG43857.1 acyl-ACP thioesterase [Iris germanica] |
| AAG43858.1 | AAG43858.1 acyl-ACP thioesterase [Iris germanica] |
| AAG43860.1 | AAG43860.1 acyl-ACP thioesterase [Iris tectorum] |
| AAG43861.1 | AAG43861.1 acyl-ACP thioesterase [Iris tectorum] |
| AAL15645.1 | AAL15645.1 palmitoyl-ACP thioesterase, partial [Elaeis guineensis] |
| AAM09524.1 | AAM09524.1 palmitoyl-acyl carrier protein thioesterase, partial [Elaeis oleifera] |
| AAN17328.1 | AAN17328.1 palmitoyl-acyl carrier protein thioesterase, partial [Elaeis oleifera] |

| | |
|---|---|
| AAQ08202.1 | AAQ08202.1 acyl-ACP thioesterase FATB [Helianthus annuus] |
| AAX51636.1 | AAX51636.1 chloroplast palmitoyl/oleoyl specific acyl-acyl carrier protein thioesterase precursor, partial [Diploknema butyracea] |
| AAX51637.1 | AAX51637.1 chloroplast stearoyl/oleoyl specific acyl-acyl carrier protein thioesterase precursor, partial [Madhuca longifolia var. latifolia] |
| ABB71579.1 | ABB71579.1 seed-specific acyl-acyl carrier protein thioesterase [Cuphea calophylla subsp. mesostemon] |
| ABB71581.1 | ABB71581.1 acyl-acyl carrier protein thioesterase [Cuphea calophylla subsp. mesostemon] |
| ABC47311.1 | ABC47311.1 FATB [Populus tomentosa] |
| ABD83939.1 | ABD83939.1 palmitoyl-ACP thioesterase [Elaeis guineensis] |
| ABD91726.1 | ABD91726.1 acyl-ACP thioesterase [Glycine max] |
| ABH11710.1 | ABH11710.1 palmitoyl-ACP thioesterase [Brassica napus] |
| ABI18986.1 | ABI18986.1 palmitoyl-ACP thioesterase [Brassica juncea] |
| ABK96561.1 | ABK96561.1 unknown [Populus trichocarpa x Populus deltoides] |
| ABL85052.1 | ABL85052.1 acyl-ATP thioesterase [Brachypodium sylvaticum] |
| ABO38554.1 | ABO38554.1 acyl ACP-thioesterase [Arachis hypogaea] |
| ABO38555.1 | ABO38555.1 acyl ACP-thioesterase [Arachis hypogaea] |
| ABO38556.1 | ABO38556.1 acyl ACP-thioesterase [Arachis hypogaea] |
| ABO38558.1 | ABO38558.1 acyl ACP-thioesterase [Arachis hypogaea] |
| ABU96744.1 | ABU96744.1 chloroplast acyl-ACP thioesterase [Jatropha curcas] |
| ABV54795.1 | ABV54795.1 acyl-ACP thioesterase [Ricinus communis] |
| ACF17654.1 | ACF17654.1 putative acyl-ACP thioesterase B [Capsicum annuum] |
| ACF79194.1 | ACF79194.1 unknown [Zea mays] |
| ACF88154.1 | ACF88154.1 unknown [Zea mays] |
| ACG29027.1 | ACG29027.1 myristoyl-acyl carrier protein thioesterase [Zea mays] |
| ACG37355.1 | ACG37355.1 myristoyl-acyl carrier protein thioesterase [Zea mays] |
| ACN27901.1 | ACN27901.1 unknown [Zea mays] |
| ACQ57190.1 | ACQ57190.1 acyl acyl-carrier-protein thioesterase type B, partial [Camellia oleifera] |
| ACQ63293.1 | ACQ63293.1 acyl acyl-carrier-protein thioesterase type B, partial [Camellia oleifera] |
| BAG90954.1 | BAG90954.1 unnamed protein product [Oryza sativa Japonica Group] |
| BAG93117.1 | BAG93117.1 unnamed protein product [Oryza sativa Japonica Group] |
| CAA54060.1 | CAA54060.1 acyl-ACP thioesterase [Cuphea lanceolata] |
| CAA85387.1 | CAA85387.1 acyl-(acyl carrier protein) thioesterase [Arabidopsis thaliana] |

| | |
|---|---|
| CAA85388.1 | CAA85388.1 acyl-(acyl carrier protein) thioesterase [Arabidopsis thaliana] |
| CAB60830.1 | CAB60830.1 acyl-(ACP) thioesterase type B [Cuphea lanceolata] |
| CAC19933.1 | CAC19933.1 thioesterase type B [Cuphea lanceolata] |
| CAC19934.1 | CAC19934.1 fatty acyl-(ACP) thioesterase type B [Cuphea lanceolata] |
| CAC80370.1 | CAC80370.1 Acyl-ACP thioesterase [Helianthus annuus] |
| CAC80371.1 | CAC80371.1 Acyl-ACP thioesterase [Helianthus annuus] |
| CAN81819.1 | CAN81819.1 hypothetical protein VITISV_008781 [Vitis vinifera] |
| CAO65585.1 | CAO65585.1 unnamed protein product [Vitis vinifera] |
| JF338903 | EAY86877.1 hypothetical protein OsI_08261 [Oryza sativa Indica Group] |
| JF338904 | EAY86884.1 hypothetical protein OsI_08268 [Oryza sativa Indica Group] |
| JF338905 | EAY99617.1 hypothetical protein OsI_21596 [Oryza sativa Indica Group] |
| JF338906 | EAZ01545.1 hypothetical protein OsI_23580 [Oryza sativa Indica Group] |
| JF338907 | EAZ37535.1 hypothetical protein OsJ_21865 [Oryza sativa Japonica Group] |
| JF338908 | EEE92766.1 hypothetical protein POPTR_0006s16060g [Populus trichocarpa] |
| EAY86877.1 | EEE92767.1 acyl-ACP thioesterase family protein [Populus trichocarpa] |
| EAY86884.1 | EEF03527.1 hypothetical protein POPTR_0018s06290g [Populus trichocarpa] |
| EAY99617.1 | EER87824.1 hypothetical protein SORBI_3010G033300 [Sorghum bicolor] |
| EAZ01545.1 | EER88593.1 hypothetical protein SORBI_3010G180400 [Sorghum bicolor] |
| EAZ37535.1 | EES06498.1 hypothetical protein SORBI_3004G088100 [Sorghum bicolor] |
| EEE92766.1 | lcl\|JF338903.1_prot_AEM72519.1_1 [gene=FatB1] [protein=acyl-ACP thioesterase FatB1] [protein_id=AEM72519.1] [location=339..1592] |
| EEE92767.1 | lcl\|JF338904.1_prot_AEM72520.1_1 [gene=FatB2] [protein=acyl-ACP thioesterase FatB2] [protein_id=AEM72520.1] [location=237..1508] |
| EEF03527.1 | lcl\|JF338905.1_prot_AEM72521.1_1 [gene=FatB3] [protein=acyl-ACP thioesterase FatB3] [protein_id=AEM72521.1] [location=194..1438] |
| EER87824.1 | lcl\|JF338906.1_prot_AEM72522.1_1 [gene=FatB1] [protein=acyl-ACP thioesterase FatB1] [protein_id=AEM72522.1] [location=56..1315] |
| EER88593.1 | lcl\|JF338907.1_prot_AEM72523.1_1 [gene=FatB2] [protein=acyl-ACP thioesterase FatB2] [frame=3] [partial=5'] [protein_id=AEM72523.1] [location=<1..1241] |
| EES06498.1 | lcl\|JF338908.1_prot_AEM72524.1_1 [gene=FatB3] [protein=acyl-ACP thioesterase FatB3] [protein_id=AEM72524.1] [location=51..1289] |

**Subfamily B**

| Acc. No | Description |
|---|---|
| ACG41291.1 | ACG41291.1 myristoyl-acyl carrier protein thioesterase [Zea mays] |
| BAD73184.1 | BAD73184.1 putative acyl-(acyl carrier protein) thioesterase [Oryza sativa Japonica Group] |
| CAN60643.1 | CAN60643.1 hypothetical protein VITISV_039528 [Vitis vinifera] |
| CAO42218.1 | CAO42218.1 unnamed protein product [Vitis vinifera] |
| CAO68322.1 | CAO68322.1 unnamed protein product [Vitis vinifera] |
| EDQ49895.1 | EDQ49895.1 predicted protein, partial [Physcomitrella patens] |
| EDQ50215.1 | EDQ50215.1 predicted protein, partial [Physcomitrella patens] |
| EDQ60538.1 | EDQ60538.1 predicted protein [Physcomitrella patens] |
| EDQ61388.1 | EDQ61388.1 predicted protein, partial [Physcomitrella patens] |
| EDQ65090.1 | EDQ65090.1 predicted protein [Physcomitrella patens] |
| EDQ70794.1 | EDQ70794.1 predicted protein, partial [Physcomitrella patens] |
| EDQ82894.1 | EDQ82894.1 predicted protein, partial [Physcomitrella patens] |
| EEE54649.1 | EEE54649.1 hypothetical protein OsJ_01925 [Oryza sativa Japonica Group] |
| EEE96114.1 | EEE96114.1 hypothetical protein POPTR_0012s04850g [Populus trichocarpa] |
| EEE96971.2 | EEE96971.2 hypothetical protein POPTR_0012s10330g [Populus trichocarpa] |
| EEF01000.1 | EEF01000.1 hypothetical protein POPTR_0010s12760g [Populus trichocarpa] |
| EEF06404.2 | EEF06404.2 hypothetical protein POPTR_0015s11170g [Populus trichocarpa] |
| EEF36100.1 | EEF36100.1 palmitoyl-acyl carrier protein thioesterase [Ricinus communis] |
| EEF51750.1 | EEF51750.1 palmitoyl-acyl carrier protein thioesterase [Ricinus communis] |
| EER96252.1 | EER96252.1 hypothetical protein SORBI_3002G111500 [Sorghum bicolor] |

**Subfamily C**

| Acc. No | Description |
|---|---|
| AAA19864.1 | AAA19864.1 oleoyl-acyl carrier protein thioesterase, partial [Coriandrum sativum] |
| AAA33019.1 | AAA33019.1 oleoyl-acyl carrier protein thioesterase [Carthamus tinctorius] |
| AAA33020.1 | AAA33020.1 oleoyl-acyl carrier protein thioesterase [Carthamus tinctorius] |
| AAB51523.1 | AAB51523.1 acyl-ACP thioesterase [Garcinia mangostana] |
| AAB51524.1 | AAB51524.1 acyl-ACP thioesterase [Garcinia mangostana] |
| AAC49002.1 | AAC49002.1 Br FatA1 [Brassica rapa] |
| AAC72883.1 | AAC72883.1 thioesterase FatA1 [Cuphea hookeriana] |
| AAG35064.1 | AAG35064.1 acyl-ACP thioesterase [Capsicum chinense] |
| AAG43859.1 | AAG43859.1 acyl-ACP thioesterase [Iris germanica] |
| AAL77443.1 | AAL77443.1 acyl-ACP thioesterase [Iris tectorum] |
| AAL77445.1 | AAL77445.1 acyl-ACP thioesterase (chloroplast) [Perilla frutescens] |
| AAL79361.1 | AAL79361.1 acyl-ACP thioesterase FATA1, partial (plastid) [Helianthus annuus] |
| AAQ08223.1 | AAQ08223.1 acyl-ACP thioesterase, partial [Helianthus annuus] |
| ABR18461.1 | ABR18461.1 unknown [Picea sitchensis] |
| ABS30422.1 | ABS30422.1 acyl-ACP thioesterase FatA [Ricinus communis] |
| ABX82799.1 | ABX82799.3 acyl-ACP thioesterase [Jatropha curcas] |
| ACF78226.1 | ACF78226.1 unknown [Zea mays] |
| ACG42559.1 | ACG42559.1 oleoyl-acyl carrier protein thioesterase [Zea mays] |
| BAF25515.1 | BAF25515.1 Os09g0505300, partial [Oryza sativa Japonica Group] |
| CAA52069.1 | CAA52069.1 acyl-ACP thioesterase [Brassica napus] |
| CAA52070.1 | CAA52070.1 acyl ACP thioesterase [Brassica napus] |
| CAA85389.1 | CAA85389.1 acyl-(acyl carrier protein) thioesterase [Arabidopsis thaliana] |
| CAB45504.1 | CAB45504.1 oleoyl-[acyl-carrier-protein] hydrolase-like protein [Arabidopsis thaliana] |
| CAC39106.1 | CAC39106.1 oleoyl hydrolase [Brassica juncea] |
| CAD32683.1 | CAD32683.1 acyl-[acyl-carrier protein] thioesterase [Triticum aestivum] |
| CAO17726.1 | CAO17726.1 unnamed protein product [Vitis vinifera] |
| CAO24254.1 | CAO24254.1 unnamed protein product [Vitis vinifera] |

| | |
|---|---|
| EAZ45287.1 | EAZ45287.1 hypothetical protein OsJ_29929 [Oryza sativa Japonica Group] |
| EEC84846.1 | EEC84846.1 hypothetical protein OsI_31951 [Oryza sativa Indica Group] |
| EEE82328.1 | EEE82328.2 hypothetical protein POPTR_0002s24700g [Populus trichocarpa] |
| EER99146.1 | EER99146.1 hypothetical protein SORBIDRAFT_02g029190 [Sorghum bicolor] |
| EER99147.1 | EER99147.1 hypothetical protein SORBI_3002G256900 [Sorghum bicolor] |

**Fat B Class I, II and III**

| Acc. No | Class | Reference |
|---|---|---|
| AAB71731.1 | Class III | |
| AAC49179.1 | Class III | |
| AAD42220.2 | Class I | |
| AAG43857.1 | Class I | |
| AAG43858.1 | Class I | |
| CnFatB1 | Class I | |
| CnFatB2 | Class I | |
| CnFatb3 | Class II | |
| CVFATB1 | Class III | |
| CVFATB2 | Class I | |
| EDQ65090.1 | Class I | |
| EER87824.1 | Class I | |
| EER88593.1 | Class I | |
| EER96252.1 | Class I | Jing F, Cantu DC, Tvaruzkova J, Chipman JP, Nikolau BJ, Yandeau-Nelson MD, et al. Phylogenetic and experimental characterization of an acyl-ACP thioesterase family reveals significant diversity in enzymatic specificity and activity. BMC Biochem. BioMed Central Ltd; 2011;12: 44. doi:10.1186/1471-2091-12-44 |
| pHA-3 | | Abrizah O, Lazarus CM, Stobart AK. Isolation of a cDNA clone encoding an awl-awl carrier protein thioesterase from the mesocarp of oil palm (Elaeis guineensis). J Oil Palm Res. 1999; 81–87. Available: http://palmoilis.mpob.gov.my/publications/99_10-p9.pdf |
| AAD28187.1 | | Asemota O, San CT, Shah FH. Isolation of a kernel oleoyl-ACP thioesterase gene from the oil palm Elaeis guineensis Jacq. Afr J Biotechnol. 2004;3: 199–201 |
