# Supplementary material for: Comparative genomic and transcriptomic analysis of selected fatty acid biosynthesis genes and CNL disease resistance genes in oil palm
Source: PLoS One. 2018 Apr 19;13(4):e0194792. doi: 10.1371/journal.pone.0194792 (PMC5908059; doi:10.1371/journal.pone.0194792)
Supplement: S1 Fig — [A] Alignment of two oil palm FATAs with orthologs from A. thaliana and Z. mays. [B] Alignment of two oil palm FATBs with orthologs from A. thaliana and Z. mays. [C] Protein sequence alignment of the orthologous cluster OG1.5_1281 of stearoyl-acyl carrier protein desaturases (SAD) aligned using MUSCLE to produce more than 70% identity between oil palm and A. thaliana and maize proteins. (PDF) [file pone.0194792.s004.pdf]

A.

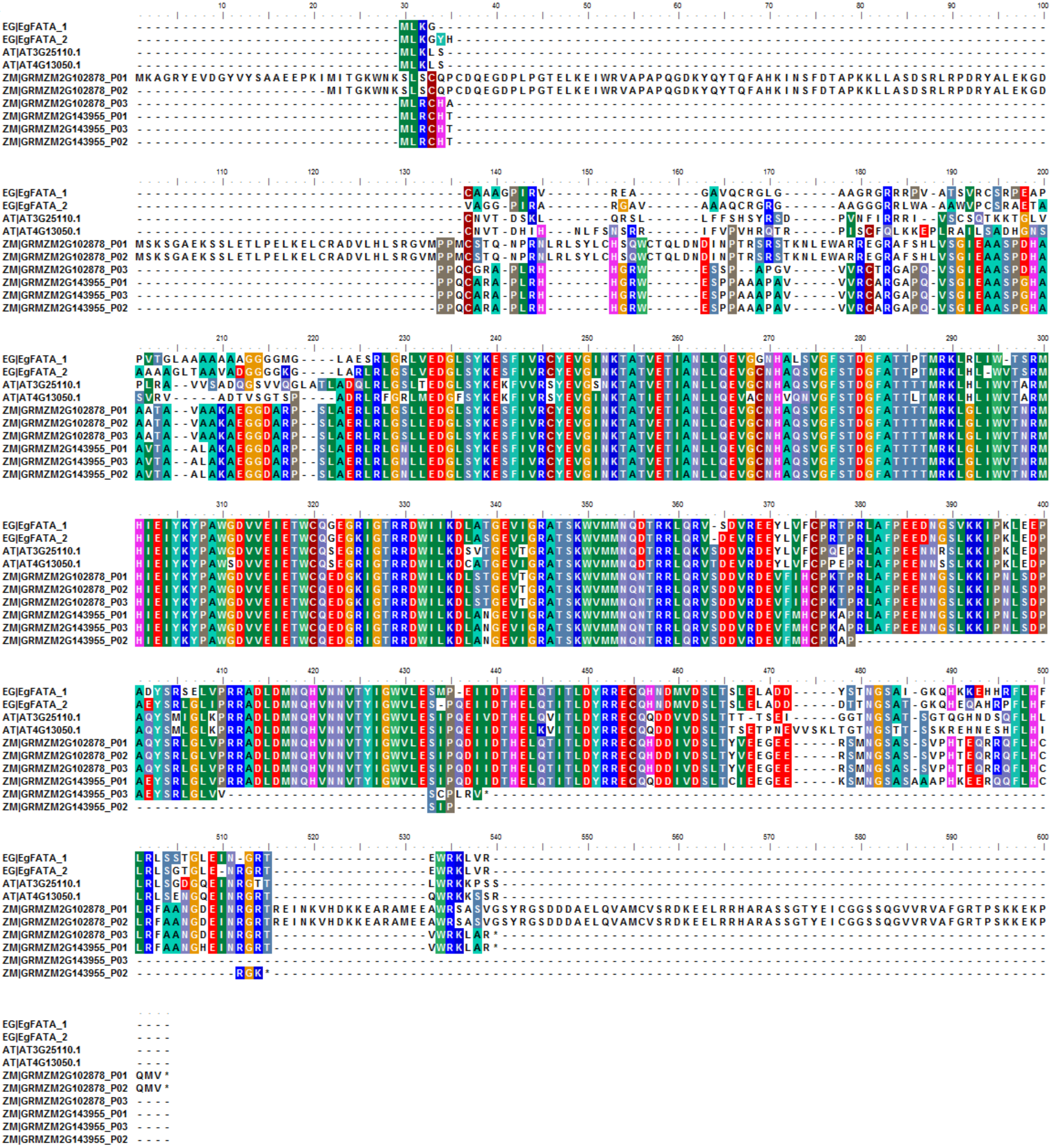
EG|EgFATA_1
EG|EgFATA_2
AT|AT3G25110.1
AT|AT4G13050.1
ZM|GRMZM2G102878_P01
ZM|GRMZM2G102878_P02
ZM|GRMZM2G102878_P03
ZM|GRMZM2G143955_P01
ZM|GRMZM2G143955_P03
ZM|GRMZM2G143955_P02

B.

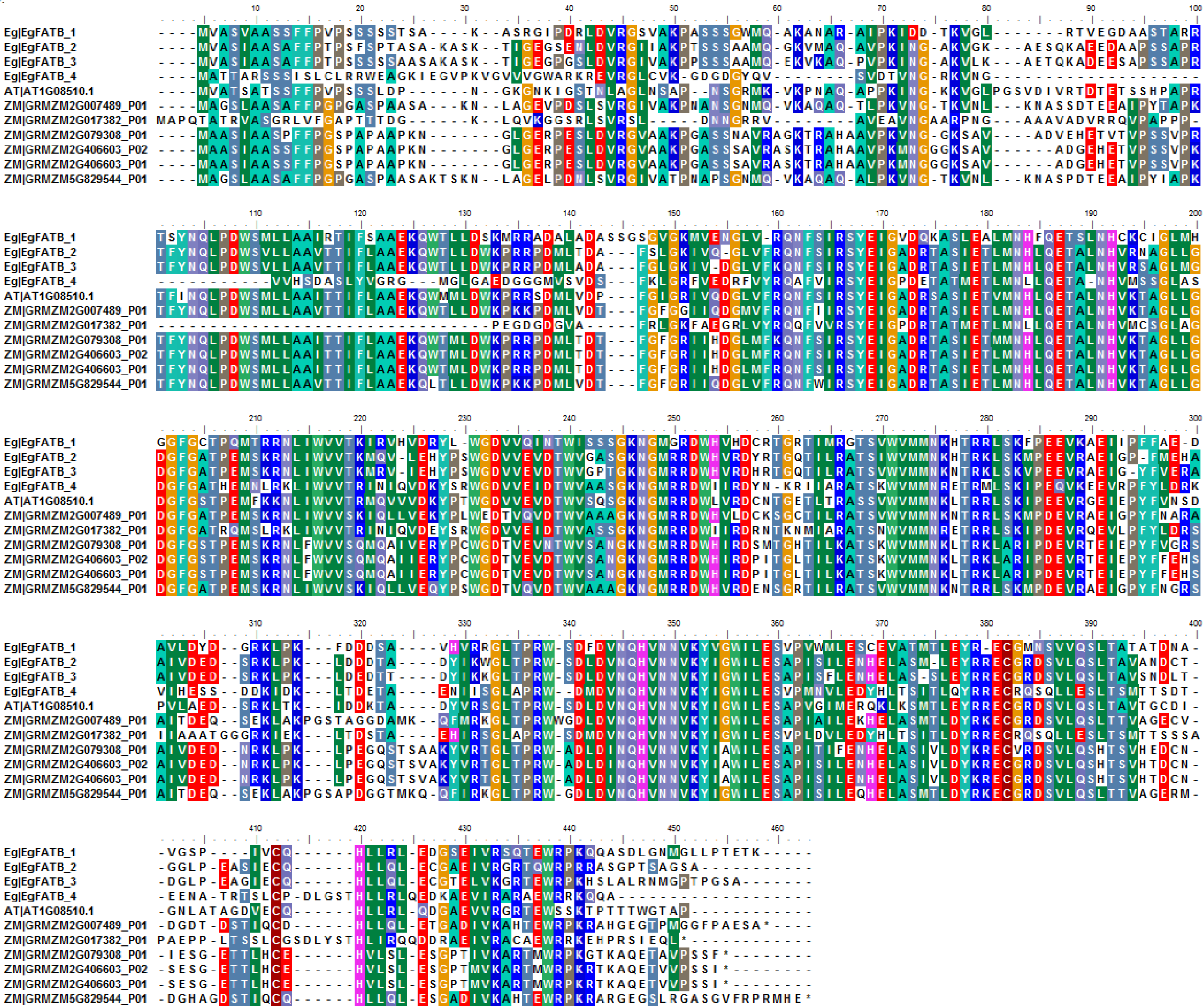
Eg|EgFATB_1
Eg|EgFATB_2
Eg|EgFATB_3
Eg|EgFATB_4
AT|AT1G08510.1
ZM|GRMZM2G007489_P01
ZM|GRMZM2G017382_P01
ZM|GRMZM2G079308_P01
ZM|GRMZM2G406603_P02
ZM|GRMZM2G406603_P01
ZM|GRMZM5G829544_P01

C.

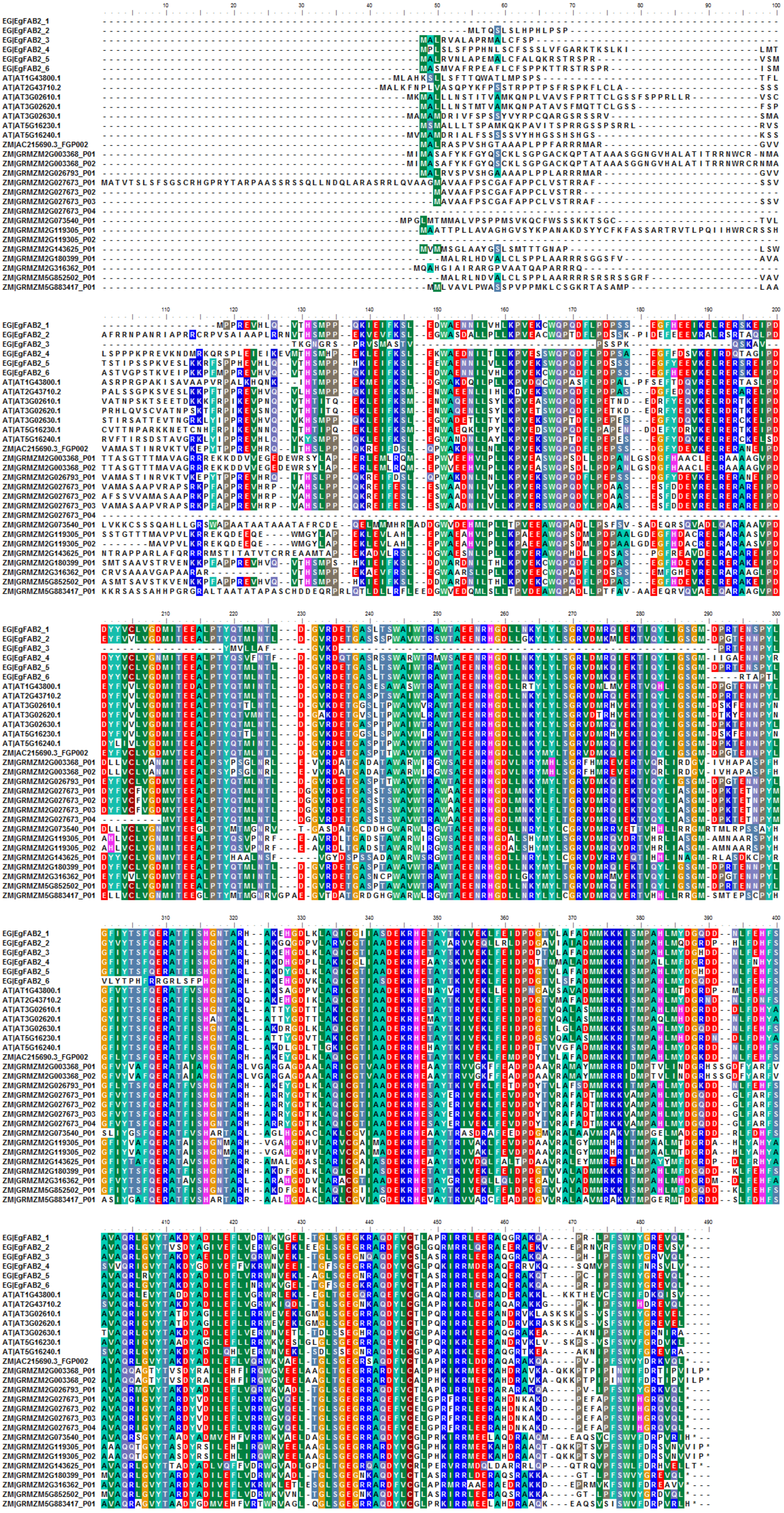
EG|EgFAB2_1
EG|EgFAB2_2
EG|EgFAB2_3
EG|EgFAB2_4
EG|EgFAB2_5
EG|EgFAB2_6
AT|AT1G43800.1
AT|AT2G43710.2
AT|AT3G02610.1
AT|AT3G02620.1
AT|AT3G02630.1
AT|AT5G16230.1
AT|AT5G16240.1
ZM|AC215690.3_FGP002
ZM|GRMZM2G003368_P01
ZM|GRMZM2G003368_P02
ZM|GRMZM2G026793_P01
ZM|GRMZM2G027673_P01
ZM|GRMZM2G027673_P02
ZM|GRMZM2G027673_P03
ZM|GRMZM2G027673_P04
ZM|GRMZM2G073540_P01
ZM|GRMZM2G119305_P01
ZM|GRMZM2G119305_P02
ZM|GRMZM2G143625_P01
ZM|GRMZM2G180399_P01
ZM|GRMZM2G316362_P01
ZM|GRMZM5G852502_P01
ZM|GRMZM5G883417_P01
